# Case report: *SLC6A1* mutations presenting with isolated absence seizures: description of 2 novel cases

**DOI:** 10.3389/fnins.2023.1219244

**Published:** 2023-06-29

**Authors:** Davide Caputo, Silvana Franceschetti, Barbara Castellotti, Elena Freri, G. Zorzi, Veronica Saletti, Laura Canafoglia, Tiziana Granata

**Affiliations:** ^1^Department of Pediatric Neuroscience, Fondazione IRCCS Istituto Neurologico Carlo Besta, Milan, Italy; ^2^Department of Neurophysiopathology, Fondazione IRCCS Istituto Neurologico Carlo Besta, Milan, Italy; ^3^Department of Diagnostic and Technology, Unit of Medical Genetics and Neurogenetics, Fondazione IRCCS Istituto Neurologico Carlo Besta, Milan, Italy; ^4^Integrated Diagnostics for Epilepsy, Department of Diagnostic and Technology, Fondazione IRCCS Istituto Neurologico Carlo Besta, Milan, Italy

**Keywords:** absence, epilepsy, *SLC6A1*, intellectual disability, GABA

## Abstract

We report the clinical and EEG data of two patients harboring heterozygous *SLC6A1* mutations, who presented with typical absence seizures at 3 Hz spike and wave as well as with mild cognitive disability. Neuroradiological and other laboratory investigations were normal. Our observations suggest that *SLC6A1* mutations can be suspected in children with typical absences as the only seizure type, especially if associated with, even mild, cognitive deficits.

## Introduction

The Solute Carrier Family 6 Member 1 (*SLC6A1*) gene encodes GAT-1, a voltage-dependent gamma-aminobutyric acid (GABA) transporter that is responsible for the re-uptake of GABA from the synapse. GABA is the principal inhibitory neurotransmitter that counterbalances neuronal excitation in the brain. Disruption of this inhibitory balance can result in seizures and other neurodevelopmental disorders (Mermer et al., 2021). To date, pathogenic variants in *SLC6A1* have been associated with a complex neurodevelopmental disorder mainly characterized by mild-to-severe developmental delay or intellectual disability, epilepsy, movement disorders, and neurobehavioral and/or psychiatric manifestations (Goodspeed et al., 2023). Seizures are among the main symptoms in almost 90% of the described patients. After initial descriptions (Dikow et al., 2014; Carvill et al., 2015), two main case series (Johannesen et al., 2018; Goodspeed et al., 2020) indicated that pathogenic variants in the *SLC6A1* gene are frequently associated with severe Epilepsy with Myoclonic-Atonic Seizures [EMAtS, previously termed Myoclonic-Astatic Epilepsy (MAE)], often preceded by developmental delay. Absence seizures are commonly reported, but usually in the context of MAE, and not as the only type of seizure (Carvill et al., 2015; Johannesen et al., 2018; Posar and Visconti, 2019; Goodspeed et al., 2020; Mori et al., 2023).

In this report, we describe the clinical and EEG data of two patients with mild cognitive disability in whom epilepsy presented with typical absences, completely controlled by anti-seizure medications.

## Case description

**Figure 1:**
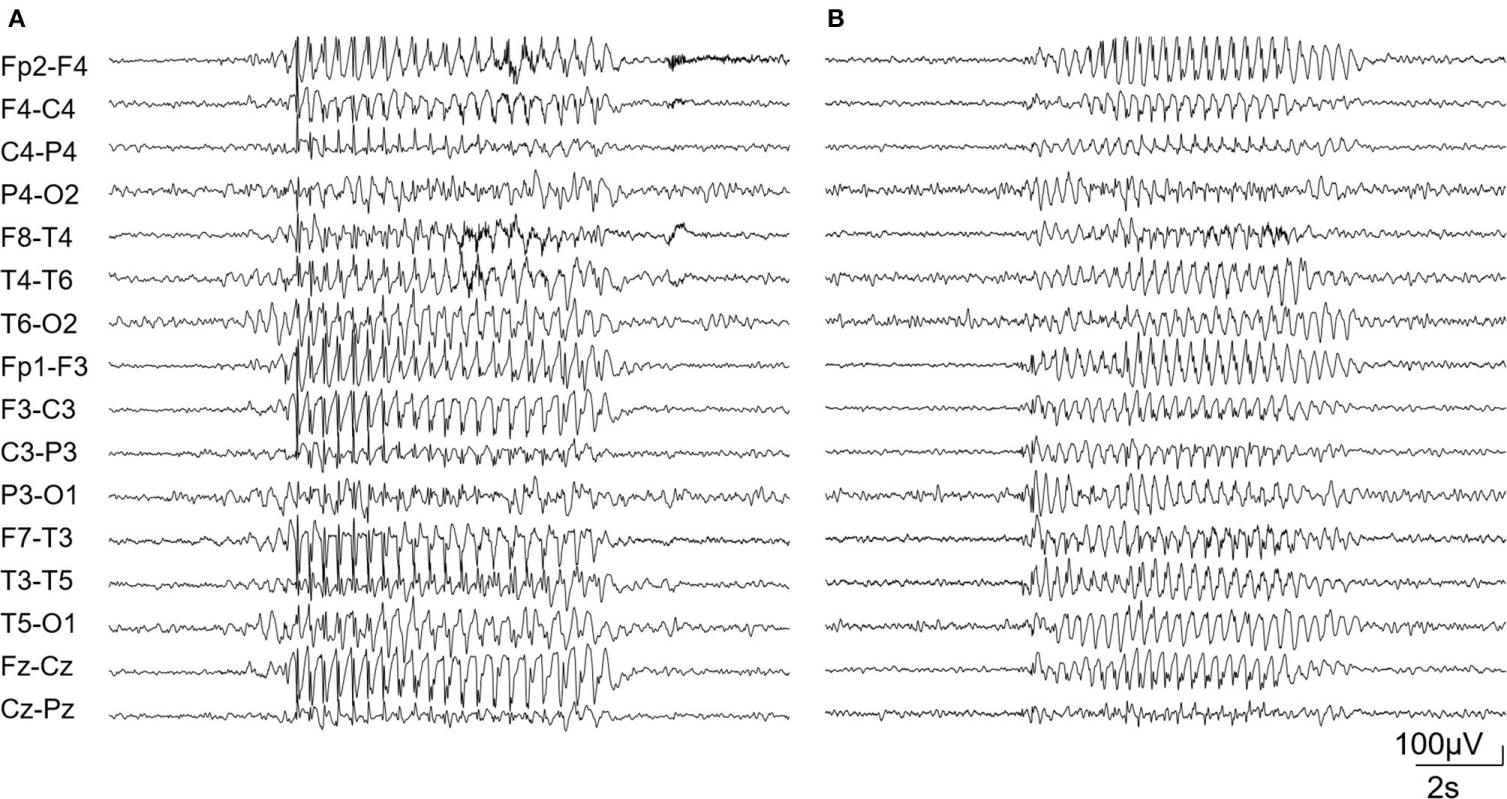
Diffuse bursts of the 3-Hz spike and wave complex in patient 1 **(A)** and 2 **(B)**.

Case 1 is a 20-year-old male. No familiar history of any neurological disease was reported. Pregnancy ended in cesarean section at 35 weeks of gestation due to premature membrane rupture. His Apgar score was 7 at 1′, 9 at 5′, and 10 at 10′. At the age of 2 years, the child was first evaluated for developmental delay. No signs suggestive of autism spectrum disorder were noticed. MRI and EEG were normal. Array CGH detected a 5p15.2 microdeletion inherited from his asymptomatic father. At the age of 5 years, the child began experiencing daily episodes of reduced awareness and mild oro-mandibular automatisms. Video EEG documented absence seizures at 3-Hz spike and wave discharges (Figure 1A). The EEG background activity and sleep organization were normal. Absences were controlled by combined valproic acid and ethosuximide treatment. Initial genetic screening, including karyotyping and *FMR1* and *SLC2A1* sequence analyses failed to detect variants. At 17 years of age, a multigene epilepsy panel (see Supplementary material) revealed a *de novo* variant in the *SLC6A1* gene (NM_003042.3): c.1648G>A/p.Gly550Arg, (rs886042046) (Mattison et al., 2018; Trinidad et al., 2022). Segregation analysis ruled out the presence of the variant in both of the healthy parents.

Case 2 is a 9-year-old boy, born at term after normal pregnancy. His Apgar score was 6 at 1′ and 9 at 5′. Family history for neurological disease was negative. Starting from the age of 12 months, a slight delay in psychomotor development was evident: the infant was able to sit unassisted at 10 months and to walk at 16 months. His Developmental Quotient, assessed using the Griffith's Scale, was 71 at the age of 12 months. At this age, the EEG was normal. The child began experiencing daily episodes of reduced awareness at the age of 5 years. EEG confirmed the presence of absence seizures associated with 3Hz spike and wave discharges (Figure 1B). Treatment with valproic acid led to seizure control. Early diagnostic investigations, including organic urinary acids, plasmatic amino acids, *FMR1* analysis, array-CGH, and MRI were normal. At 6 years old, the Wechsler Intelligence Scale for Children (WISC-IV) showed a mild intellectual disability (total IQ = 67). No signs suggestive of autism spectrum disorder were noticed. At 7 years of age, a multigene epilepsy panel (see Supplementary material) showed the variant (NM_003042.3): c.1084G>A/p.Gly362Arg (rs1131691302) in the *SLC6A1* gene (Johannesen et al., 2018). Variant segregation in family members was not possible. The clinical and genetic data of the two patients are summarized in Table 1.

**Table 1:** Clinical aspects of the two patients described.

	**Sex, years**	**Age at epilepsy onset (years)**	**Anti-seizure Medications**	***SLC6A1* variant**	**References**
Case 1	M, 20	5	VPA, ETS	p.Gly550Arg	Mattison et al., 2018
Case 2	M, 9	5	VPA	p.Gly362Arg	Johannesen et al., 2018

VPA, valproic acid; ETS, ethosuximide.

## Discussion

Our report shows that mutations in the *SLC6A1* gene may lead to typical absence seizures associated with mild intellectual disability. The association between epilepsy and developmental delay/cognitive disability is known to occur in *SCL6A1* patients; absences are the most frequently reported seizure in these patients (Johannesen et al., 2018; Goodspeed et al., 2020); however, they have been mostly reported in the context of EMAtS or in the context of epilepsy that did not fit codified epileptic syndromes.

The presence of seizures in patients carrying *SLC6A1* mutations is likely related to impairment of inhibitory neurotransmission induced by the genetic anomaly. *SCL6A1* encodes GAT-1, one of the major GABA transporters in the brain, responsible for the re-uptake of GABA from the synapses. This hypothesis is supported by experimental models that demonstrate dysfunctional GABA transmission in spontaneous or induced rodent models of absence epilepsy, with spike-wave discharges. Moreover, Cope et al. (2009) found that *Slc6a1*-knockout mice develop spike-wave discharges characteristic of absence seizures. The pathophysiological mechanism that generates spike and wave discharges involves a complex circuitry that includes the thalamus, cortex, and basal ganglia. The loss of GAT-1 function leads to higher levels of GABA in the thalamus, but not in the cortex. The resulting enhanced tonic GABA-A inhibition in both thalamo-cortical neurons (see Crunelli et al., 2020 for a review) and ventrobasal thalamo-cortical neurons is sufficient to elicit absence seizures even in wild-type rodents (Trinidad et al., 2022). The complexity of the network may explain the variable seizure phenotype and severity, ranging from pure absences to more severe EMAts encephalopathy, as well as the variable degree of cognitive impairment (Goodspeed et al., 2023).

The p.Gly550Arg variant, detected in our case 1, has been described in a patient with generalized epilepsy and absence and tonic-clonic seizures (Mattison et al., 2018). The p.Gly362Arg variant, detected in our case 2, has been reported in two cases (Johannesen et al., 2018): a 1-year old child with Lennox-Gastaut syndrome and a child with temporal lobe epilepsy. We cannot find a reason for the extreme phenotypical variability associated with *SLC6A1* variants, we can only hypothesize on the influence of genetic background and epigenetic factors.

Our data suggest that comprehensive genetic evaluation, including molecular analysis for monogenic conditions associated with epilepsy, is warranted in children with typical absences associated with intellectual disability.

## Data availability statement

The original contributions presented in the study are included in the article/Supplementary material, further inquiries can be directed to the corresponding author.

## Ethics statement

Written informed consent was obtained from the individual(s), and minor(s)' legal guardian/next of kin, for the publication of any potentially identifiable images or data included in this article.

## Author contributions

DC and SF contributed to the preparation of the original draft. GZ and VS were involved in the patient treatment. BC performed genetic analysis. LC, EF, and TG reviewed the manuscript. All authors contributed to the writing of the final manuscript.
