# Supplementary material for: Case report: SLC6A1 mutations presenting with isolated absence seizures: description of 2 novel cases
Source: Front Neurosci. 2023 Jun 29;17:1219244. doi: 10.3389/fnins.2023.1219244 (PMC10339345; doi:10.3389/fnins.2023.1219244)
Supplement: Supplementary file 1 [file Data_Sheet_1.docx]

Methods used for the genetic testing:

Next Generation Sequencing (NGS) analysis by multigenic target panel was performed.

The panel was designed including 340 genes related to developmental and epileptic encephalopathy (see below for the list of genes). The average coverage of this panel at 20X read depth is found to be 99.5%. A study of CNVs (copy number variation) that excluded the presence of alterations in gene dosage was also performed.

List of gene included in the panel:

*AARS1, ABAT, ACTB, ACTG1, ADAR, ADGRG1, ADPRHL2, ADSL, AFG3L2, AGA, AKT3, ALDH5A1, ALDH7A1, ALG10, ALG11, ALG13, ALG6, ALG8, AMACR, AMT, ANKRD11, AP2M1, AP3B2, APBA2, ARFGEF2, ARG1, ARHGEF9, ARV1, ARX, ATP1A1, ATP1A2, ATP1A3, ATP6AP2, BRAT1, BTD, CABP4, CACNA1A, CACNA1B, CACNA1E, CACNA1H, CACNA2D2, CACNB4, CAD, CASK, CASR, CDK19, CDKL5, CERS1, CHD2, CHRNA2, CHRNA4, CHRNB2, CIC, CLCN2, CLCN4, CLN3, CLN5, CLN6, CLTC, CNTNAP2, COL4A1, COL4A2, CPA6, CPT2, CRH, CSNK2B, CSTB, CTNNA2, CTSD, CUL4B, CUX2, DCHS1, DCX, DEAF1, DENND5A, DEPDC5, DHDDS, DHPS, DIAPH1, DNM1, DOCK7, DPYD, DYNC1H1, EEF1A2, EFHC1, EMX2, EPM2A, ERMARD, ETFA, ETFB, ETFDH, FAT4, FGF12, FH, FIG4, FLNA, FOLR1, FOXG1, FOXP1, FOXP2, FRRS1L, GABRA1, GABRA2, GABRA5, GABRB2, GABRB3, GABRD, GABRG2, GAMT, GBA, GCDH GCH1, GJA1, GLDC, GLI3, GLS, GNAO1, GNAQ, GNB5, GNE, GOSR2, GPHN, GPSM2, GRIA3, GRIA4, GRIN1, GRIN2A, GRIN2B, GRIN2D, HCN1, HCN2, HCN4, HECW2, HEPACAM, HESX1, HNRNPU, IQSEC2, IRF2BPL, ITPA, KATNB1, KCNA1, KCNA2, KCNB1, KCNC1, KCND2, KCNE2, KCNH1, KCNH5, KCNH8, KCNJ10, KCNJ6, KCNK4, KCNMA1, KCNQ2, KCNQ3, KCNQ5, KCNT1, KCNT2, KCNV2, KCTD3, KCTD7, KDM6A, KIF1A, KIF2A, KIF5A, KIF5B, KIF5C, KMT2D, L2HGDH, LAMA2, LAMB1, LAMC3, LGI1, LIAS, MAGI2, MAPK10, MBD5, MECP2, MED17, MEF2C, MOCS1, MTHFR, MTHFS, MTOR, NACC1, NAXD, NAXE, NDE1, NDP, NECAP1, NEDD4L, NEU1, NEXMIF, NHLRC1, NPC1, NPC2, NPRL2, NPRL3, NRXN1, NSDHL, NTRK2, NUS1, OCLN, PAFAH1B1, PARS2, PCDH19, PDE2A, PEX5L, PGK1, PHGDH, PIGA, PIGB, PIGN, PIGO, PIGQ, PIGT, PIK3CA, PIK3R2, PLCB1, PNKP, PNPO, POLG, PPP2CA, PPT1, PRICKLE1, PRNP, PRODH, PRRT2, PTEN, PTS, PURA, PYCR2, QARS, QDPR, RELN, RHEB, RHOBTB2, RNASEH2A, RNF13, ROGDI, RORA, RTTN, RYR3, SACS, SAMHD1, SCARB1, SCARB2, SCN1A, SCN1B, SCN2A, SCN3A, SCN8A, SCN9A, SEMA6B, SERPINA1, SETBP1, SIK1, SLC12A5, SLC12A6, SLC13A5, SLC19A3, SLC1A2, SLC20A2, SLC25A12, SLC25A22, SLC2A1, SLC35A2, SLC35A3, SLC46A1, SLC6A1, SLC6A8, SLC7A6OS, SLC9A6, SMARCC2, SMC1A, SMS, SNAP25, SNAP29, SPATA5, SPTAN1, SPTBN2, SRPX2, ST3GAL3, ST3GAL5, STXBP1, SUOX, SYN1, SYNGAP1, SZT2, TBC1D20, TBC1D24, TBL1XR1, TCF4, TMEM106B, TMEM63B, TNK2, TPP1, TREX1, TSC1, TSC2, TSEN15, TSEN2, TSEN54, TUBA1A, TUBA8, TUBB, TUBB2A, TUBB2B, TUBB3, TUBB4A, TUBG1, TWNK, UBE2A, UBE3A, VLDLR, WASF3, WDR4, WDR45, WDR62, WWOX, YWHAG, ZEB2*
